# Fast-Food Dietary Pattern Is Linked to Higher Prevalence of Metabolic Syndrome in Older Canadian Adults

**DOI:** 10.1155/2021/5712844

**Published:** 2021-10-21

**Authors:** Zeinab Hosseini, Mehdi Rostami, Susan J. Whiting, Hassan Vatanparast

**Affiliations:** ^1^College of Pharmacy and Nutrition, University of Saskatchewan, Saskatoon, Saskatchewan, Canada; ^2^College of Kinesiology, University of Saskatchewan, Saskatoon, Saskatchewan, Canada; ^3^Biostatistics Division, Dalla Lana School of Public Health, Health Sciences Building, University of Toronto, Toronto, Ontario, Canada

## Abstract

**Background:**

Metabolic syndrome (MetS) is known to increase the risk of cardiovascular diseases and diabetes. Diet is a key factor in prevention and development of MetS. This study aimed to determine the association between dietary patterns and MetS among Canadians 12–79 years old using the Canadian Health Measures Survey (CHMS) combined Cycles 1 and 2 data from 2007–11. We hypothesized that MetS varies among different sociodemographic and lifestyle factors and that Canadians who have less healthy dietary patterns are more likely to have MetS.

**Methods:**

In the CHMS, MetS was determined using objective health measures. The principal component analysis method was used to determine the dietary patterns. Using logistic regression, the association between MetS and dietary patterns, controlling for potential covariates, was investigated for age groups of 12–19, 20–49, and 50–79 years. Survey data were weighted and bootstrapped to be representative at the national level.

**Results:**

The prevalence of MetS was 16.9% for ages 12–79 y (*n* = 4,272, males = 49.6%), representing 26,038,108 Canadians aged 12–79 years. MetS was significantly different across sociodemographic variables; Canadians with less education, income, and activity had higher MetS prevalence than their counterparts. In older adults (50–79 years of age), the “fast-food” dietary pattern was associated with 26% (odds ratio = 1.26; 95% CI: 1.04 to 1.54; *p*=0.0195) higher likelihood of having MetS.

**Conclusions:**

Among older Canadians, MetS is associated with a “fast-food” dietary pattern after adjustment for socioeconomic/lifestyle factors. Findings suggest the importance of diet quality/composition in the development of MetS among older Canadians and the need for further longitudinal studies on MetS and diet across the lifespan.

## 1. Introduction

Cardiovascular diseases (CVDs) and diabetes are leading causes of death worldwide and in Canada [1]. To reduce the incidence of these diseases, we need to understand the underlying related risk factors. The metabolic syndrome (MetS) includes five components, which are risk factors related to CVD and diabetes [2, 3]. Based on the joint statement by the International Diabetes Federation and the American Heart Association/National Heart, Lung, and Blood Institute having three or more of these CVD-related risk factors including abdominal obesity, hyperglycemia, hypertriglyceridemia, lowered high-density cholesterol, and hypertension indicates the presence of MetS in an individual [2].

Research has shown that diet is a key factor that can contribute to the development or prevention of MetS. For metabolic disorders such as MetS, studies have indicated that assessing the diet using a holistic approach such as dietary pattern analysis may be more useful than investigating individual dietary components [4]. In most population-based studies that have evaluated the association of MetS and dietary patterns, the “Western” dietary pattern has been shown to increase the risk of MetS [5–7]. Moreover, while the “healthy” dietary pattern showed to have an association with MetS only in a few studies [5–7], rather more “heart-healthy” specific diets such as the Mediterranean diet have been found to reduce the risk of MetS in most population-based studies [7–9]. In fact, recently, dietary guidelines have been providing recommendations on adhering to diets such as the Mediterranean diet, Dietary Approaches to Stop Hypertension, and vegetarian-based dietary patterns to prevent or manage components of MetS among the population [10, 11].

The link between diet and MetS varies among different populations [7, 12, 13], and understanding this link will provide evidence for policy makers and researchers to design and implement suitable interventions for the population at high risk of diabetes and CVD. The link between dietary patterns prevalent among Canadians and MetS has not been investigated previously. The Canadian Health Measures Survey (CHMS) is a nationally representative survey conducted for the first time in Canada that includes objective health and nutrition data [14]. The aim of this study is to evaluate the status of MetS in Canada by sociodemographic and lifestyle factors and to determine the association between prevalent dietary patterns and MetS using the CHMS. We hypothesized that the prevalence of MetS is higher in Canadians with lower income and education level compared to higher and Canadians who are inactive and smokers compared to those active and nonsmokers. Also, Canadians who have healthier dietary patterns are less likely to have MetS.

## 2. Methods and Materials

### 2.1. Participants and Study Design

The CHMS is a cross-sectional nationally representative health survey that runs in biyearly cycles throughout Canada [14]. This survey is conducted by Statistics Canada in collaboration with Health Canada and the Public Health Agency of Canada. The main aim of this survey is to fill the gaps in Canadian health information. Health Canada's Research Ethics Board has obtained ethics approval of this survey. Respondents submit written consent before their participation [14]. Initially, an interview is held at the household using the “household questionnaire” to collect self-reported sociodemographic, lifestyle, and thorough health information. A few days after the household interview, the participant visits the mobile examination center for another thorough interview using the “clinic questionnaire” and to give urine and blood samples [14, 15]. Cycle 1 of this survey was conducted starting from 2007 and ending in 2009 and included approximately 5,600 participants aged 6 to 79 years; cycle 2 ran starting from 2009 and ended in 2011 and included approximately 6,400 participants ages 3 to 79 years [14, 15]. This survey was designed to cover up 96% of the target population [14]. Canadians living on reserves/remotes or in other Aboriginal settlements, institutionalized residents, and Canadian Forces full-time members were excluded from this survey [14]. The CHMS was conducted via a multistage sampling procedure, including the sampling of “collection sites” (geographical units), “dwellings,” and finally, at the level of individuals residing in the dwellings [14]. The combined “fasted subsample” data file (*n* = 5,427 respondents had fasted for a minimum of 10 hours) with a response rate of 46.3% [14] was used for the analysis of the present study. Ages 12–79 years, nonpregnant with no diagnoses of diabetes, were included in this study. The later exclusion criterion was based on previous findings indicating that since those with diagnosed diabetes tend to modify their diet based on recommendations they receive from health professionals, they should be removed when investigating the link of diet and chronic diseases [16]. There was a total of 221 [17] and 275 [18] participants with diagnosed diabetes in Cycles 1 and 2 of CHMS, respectively. The total number of the study sample used in the analysis was 4,272 that represented 26,038,108 Canadians (12–79 years) (Figure S1).

### 2.2. Dietary Assessment

We used the usual dietary intake data collected using a food frequency questionnaire through the CHMS household questionnaire [14, 15, 19]. This food frequency questionnaire was used to collect usual daily dietary intake. A total of 32 questions from seven food groups of CHMS were included in our analysis (Supplementary Table S1). The seven groups were as follows: meat; milk and dairy products; grains; fruit and vegetables; dietary fat; and water and soft drink consumption [15]. The 32 questions on the frequency of the intake from food items were used in the dietary pattern analysis of our study to compare the diet between individuals with and without MetS.

### 2.3. Metabolic Syndrome

The MetS was defined differently for ages 12–15 years and 16 years and above. For ages 12–15 years, the 2007 International Diabetes Federation-proposed cutoff (using percentiles) was used to determine MetS [20], and for those 16–79 years, having at least three out of the five components of MetS including elevated waist circumference; fasting plasma glucose (≥5.6 mmol/L); systolic or/and diastolic blood pressure (systolic ≥130 and/or diastolic ≥85 mm Hg) or drug treatment for hypertension; triglycerides (≥150 mg/dL (1.7 mmol/L)); and reduced high-density lipoprotein cholesterol (<40 mg/dL (1.0 mmol/L) in males; < 50 mg/dL (1.3 mmol/L) in females) would define the presence of MetS [3]. In addition, we used the ethnic/country-specific waist circumference cutoffs for the abdominal obesity component of MetS recommended by Alberti et al. [3].

Blood pressure reported in the CHMS was the average of five series of measurements taken with one-minute intervals following a five-minute resting period. The blood pressure was taken using a validated electronic automated oscillometric device (BpTRU™ Medical Devices Ltd., Coquitlam, British Columbia) recommended by the Canadian Hypertension Education Program and Committee of Hypertension and Survey Experts [21]. Waist circumference was measured using the World Health Organization and the National Health Institute protocols [22]. A phlebotomist drew blood from the median cubital or cephalic veins of the left arm using a standardized venipuncture technique [14]. The lab measurements used for this study were blood measurements of glucose, insulin, and lipids. For more details and information regarding health measurements, please refer to the CHMS User Guide [14, 15].

### 2.4. Socioeconomic and Lifestyle Characteristics

Factors including age, sex, income, education, physical activity, smoking, alcohol intake, ethnicity, and having a family doctor were classified into their corresponding categories. For the age variable, the specific categories were developed including 12 to 19, 20 to 49, and 50 to 79 years. The household income variable included the lowest, lower-middle, upper-middle, and highest income levels. These levels were based on the amount of household income that was self-reported by respondents: lowest income < $15,000 if 1-2 people or < $30,000 if more than 4 people were in the household; lower-middle income level included those having a total income of $15,000–$29,999 if 1-2 people; $20,000–$39,999 if 3-4 people; and $30,000–$59,999 if more than four people were living in the household; upper-middle category indicated an income of $30,00–$59,999 if 1-2 people; $40,000–$79,999 if 3-4 people; and $60,000–$79,999 if there were more than four in the household; highest-level income included those with an income of $60,000 or more if 1-2 people and an income of $80,000 or more if more than two people were living in the household [14]. Also, the education variable was categorised as less than secondary, secondary, other postsecondary, and postsecondary graduate levels. In this survey, respondents were asked questions on the type of physical activities, frequency (number of times participated), and average duration (time spend on each occasion) they had participated in these activities during the past three months [18]. The Physical Activity Index [23] was used to determine the total daily leisure-time energy expenditure. Thus, physical activity was categorised into inactive (0≤ daily energy expenditure (DEE) < 1.5), moderate activity (1.5 ≤  DEE <3), and active (DEE ≥ 3) levels [15, 24]. Alcohol intake had two categories of “ever” drinker and “never” drinker. Smoking was categorized into current smoker, former smoker, and nonsmoker categories, and self-reported ethnicity included the following five categories: white; Aboriginal, Middle East, Mediterranean, sub-Saharan African, and West Asian; Asian, South Asian, and Latin American; and Chinese and Japanese [14, 15] (ethnicity variable used in models only). Having a family doctor was also included in the analysis as yes or no.

### 2.5. Statistical Analysis

Statistics Canada provided instructions on combining the first two cycles [22]. To create the final dataset, data manipulation, cleaning, grouping, and creating the variables of interest were performed. The prevalence of MetS and its components were determined across socioeconomic and lifestyle factors as frequencies (% (standard error (SE)), two-sided 95% confidence intervals (CI)). Simple binary logistic regression was used to estimate the significant difference of MetS prevalence among different categories of the aforementioned variables. The dietary intake was determined as means ± SE and 95% CI, and the significance was determined by nonoverlapping CI of the means between the MetS group and the group without MetS. According to Statistics Canada's recommendations, the degree of freedom of 24 was used for the combined data (11 degrees of freedom from cycle 1 in addition to 13 degrees of freedom from cycle 2) [22]. Missing data on some of the variables of interest were included in the descriptive analysis (please refer to the CHMS User Guide for further explanation on nonparticipation) [15]. Alpha was set at 0.05 to detect statistically significant differences. SAS for Windows software (release 9.4, SAS Institute, Cary, North Carolina, U.S.) was used for all statistical analysis.

The dietary pattern analysis was conducted for each age group of 12–19, 20–49, and 50–79 years, separately. In order to extract dietary patterns from 32 food groups, the principle component analysis (PCA, PROC FACTOR) method was used. Using this method, components were produced by grouping correlating food intake variables in order to derive uncorrelated variables (components). Four dietary patterns were retained for each age group based on the scree-plot method [25, 26]. The Varimax rotation method was used to obtain more interpretable results [26]. These factors explained about 27, 28, and 25% of the variation in the data for ages 12–19, 20–49, and 50–79 years, respectively. The names of the dietary patterns were chosen based on the content of the foods with a factor loading cutoff of ±0.25.

Logistic regression was used to assess the magnitude of the association between MetS and the four dietary patterns extracted from dietary intake data. The multivariate adjusted odds ratio (OR) and two-sided 95% confidence intervals (CIs) were used to report the association between MetS and dietary patterns. Odds ratio is compared to those that have 1 SD lower score from the corresponding dietary pattern. Two models were used: model I adjusting for age and sex and model II adjusting for age, sex, income, education, physical activity, alcohol intake, ethnicity, smoking, and other dietary patterns 1 to 4. Weighting and bootstrapping were performed to be able to report nationally representative results.

## 3. Results

**Figure 1:**
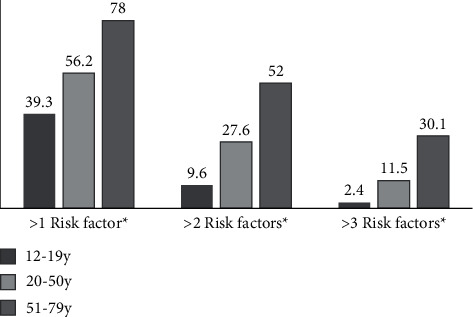
The prevalence of Canadians (12–79 y of age) with at least one, two, and three components of metabolic syndrome across the three age groups of 12–19, 20–50, and 51–79 years. Canadian Health Measures Survey combined Cycles 1 and 2, 2007–2011 (*n* = 4,272, males = 49.6%, representative of 26,038,108 Canadians aged 12 to 79 years), was conducted. ^*∗*^Significance differences between MetS prevalence of the three age groups.

The sample from CHMS combined Cycles 1 and 2 (*n* = 4,272, males = 49.6%) was representative of 26,038,108 Canadians aged 12 to 79 years (nonpregnant and with no diagnosis of diabetes). Based on this study, MetS was prevalent among 16.9% of Canadians aged 12–79 years. The prevalence was 2.4% for 12–19 years, 11.5% for 20–50 y, and 30% for 51–79 y. The most prevalent component of MetS among Canadians was abdominal obesity (32.5%) followed by reduced high-density cholesterol (27.7%) and elevated blood pressure, triglycerides, and fasting plasma glucose (23.1, 22.6, and 13.7%, respectively). Furthermore, 62% of the total population had at least one component of MetS present, and 34% had at least two components of MetS present. The prevalence of people with one, two, and three components of MetS by age groups is presented in Figure 1, indicating a significant difference among age groups in all three categories (*p* < 0.005). Compared to people without MetS, individuals with MetS were older (39 versus 52 years, *p* < 0.001) and had higher body mass index (25.14 versus 32.31 kg/m^2^ (*p* < 0.001), waist-to-hip ratio (0.88 versus 0.98, *p*=0.006), weight (71.2 versus 93.06 kg, *p* < 0.001), and Homeostatic Model Assessment of Insulin Resistance (1.78 versus 4.26, *p* < 0.001). The population with lower-middle income (25.8%) had higher MetS prevalence compared to the upper-middle income level (14.1%, *p*=0.031) (Table 1). In addition, those with some postsecondary (12.2%, *p*=0.003) and postsecondary graduation (14.2%, *p*=0.001) education levels had lower prevalence of MetS compared to those in the less than secondary (36.2%) education level group.

Physically active Canadians had a lower prevalence of MetS (9.3%) compared to moderately active (18.7%, *p*=0.002) and inactive Canadians (19.8%, *p* < 0.001). In addition, former smokers (24%) had higher MetS prevalent among them compared to nonsmokers (13%, *p*=0.002). Canadians with MetS had fewer intakes from nuts, eggs, pasta, and sport drinks and more intakes from diet soft drinks, compared to the rest of the population. No other significant differences were observed in the intake from food groups between the MetS and non-MetS categories (Supplementary Table S2).

### 3.1. Dietary Patterns

The four extracted dietary patterns and the corresponding factor loadings of food/food groups on the patterns of our sample are presented in Table 2. For ages 12–19 years, the first dietary pattern was the “Western” with positive loadings of red meat, hotdogs, sausage/bacon, chips, fries, diet soft drinks, regular soft drinks, and sport drinks. The second dietary pattern was named the “healthy-like” with positive loadings of fruit, tomato/tomato sauce, “other” vegetables (i.e., vegetables other than tomato, lettuce, green leafy salad, spinach/mustard greens/collards, and potatoes), yoghurt, diet soft drinks, and negative loadings of regular soft drinks and fruit juice. The third dietary pattern was the “salad and condiments” with positive loadings of tomato/tomato sauce, lettuce/green vegetables, spinach/mustard greens/collards, “other” vegetables (i.e., vegetables other than tomato, lettuce, green leafy salad, spinach/mustard greens/collards, and potatoes), and salad dressing/mayonnaise, and the last dietary pattern was the “protein/rice” with positive loading of red meat, nonliver organ meat, beans, nuts, eggs, spinach/mustard greens/collards, and rice.

For ages 20–49 years, the first dietary pattern was the “Western” with positive loadings of red meat, hotdogs, sausage/bacon, eggs, baked/boiled/mashed potatoes, chips, fries, white bread, pasta, and diet soft drinks. The second dietary pattern was named the “healthy-like” with positive loadings of nuts, fruits, tomato/tomato sauce, lettuce/green vegetables, spinach/mustard greens/collards, “other” vegetables (i.e., vegetables other than tomatoes, lettuce, green leafy salad, spinach/mustard greens/collards, and potatoes), yoghurt, and salad dressing/mayonnaise. The third dietary pattern was the “nuts, fruits and vegetables, dairy, and cereal” with positive loadings of nuts, fruit, other vegetables, milk, yoghurt, and cereal, and the last dietary pattern was the “organ meat” dietary pattern with positive loading of liver and other organ meat.

For ages 50–79 years, the first dietary pattern was the “healthy-like” with positive loadings of nuts, fruit, lettuce/green vegetables, spinach/mustard greens/collards, “other” vegetables (i.e., vegetables other than tomatoes, lettuce, green leafy salad, spinach/mustard greens/collards, and potatoes), cheese, yoghurt, diet soft drinks, and vegetable juice and negative loadings of regular soft drinks. The second dietary pattern was named the “salad and condiments” with positive loadings of tomato/tomato sauce, lettuce/green vegetables, and salad dressing/mayonnaise. The third dietary pattern was the “fast food” with positive loadings of hotdogs, sausage/bacon, chips, fries, and diet soft drinks, and the last dietary pattern was the “meat and potato” with positive loading of red meat, sausage/bacon, eggs, baked/boiled/mashed potatoes, and ice cream/frozen yoghurt.

### 3.2. Association between Metabolic Syndrome and Dietary Patterns

Our results showed that, after adjusting for all sociodemographic and lifestyle factors among ages 50–79 years, the “fast-food” dietary pattern was associated with 27% (OR = 1.27; 95% CI: 1.04 to 1.54; *p*=0.0195) higher likelihood of having MetS (Table 3) compared to those with 1 SD lower scores of the “fast-food” dietary patterns. No significant association was observed between MetS and other dietary patterns for younger age groups.

## 4. Discussion

This is the first study evaluating the association of MetS and dietary patterns among Canadians 12–79 years of age. The MetS prevalence was 16.9% among Canadians 12–79 years of age with abdominal obesity being the highest prevalent component of MetS. As we hypothesized, MetS was significantly different across levels of sociodemographic and lifestyle variables including age, income, education, smoking, having a family physician, and physical activity. The “fast-food” dietary pattern prevalent among the older adult Canadian population increased the risk of MetS which partly supports our hypothesis.

### 4.1. Metabolic Syndrome and Its Components among Canadians

The MetS prevalence found in our study was lower than that in a study that had used only CHMS Cycle 1 data (16.9 versus 18.3%) [27]. The lower rate in the present study could be due to the excluded people with diagnosed diabetes, which may highly likely have MetS. Based on our results, the prevalence of MetS among the adolescent population was 2.4% which is in agreement with the Canadian study using similar criteria for adolescents (2.1%) [28] with slight difference due to age range (present study including ages 12–19 years that are slightly older than their [28] age category of 10–18 years, which included ages 10 and 11 years and have not included 19-year-old participants).

### 4.2. Metabolic Syndrome and Socioeconomic and Lifestyle Factors in Canada

Our findings are in agreement with previous research investigating the link between MetS and socioeconomic factors [29–31]. Health and lifestyle behaviors; phycological; social; neighborhood; and health-care access have been indicated as factors that contribute to the link between MetS and socioeconomic factors [31]. Regarding income levels, we found that MetS is most prevalent among lower income levels compared to those with higher income. Similar results were found in other studies for both sexes [27] and in a study on women [30]. A lower income status contributes to food choices that are characterized by high energy-dense content and lower cost [30] leading to food insecurity, especially when living in poor neighborhoods with food deserts [32] and perhaps less feasible environments for physical activity [33] contributing to higher risk for metabolic disorders [30]. Our results indicate that there is less prevalence of MetS among individuals who have a higher level of education compared to those with lower levels that is in agreement with other studies [30, 31]. Research has shown that not only higher education level provides more knowledge that can be helpful in making healthier nutritional and lifestyle choices [30]; it also reflects the psychosocial contributors including depression, employment, and poverty, which are linked to obesity and poor metabolic outcomes [31].

The lower MetS prevalence among the physically active group compared to the moderately active and inactive groups was also observed in a study using Canadian data to assess the moderate-to-vigorous physical activity measured by using 7-day Actical accelerometers [34]. These researchers found that physical activity has a strong association with the risk of MetS among Canadians 18–64 years of age. Physical activity not only affects the amount of body fat mass but also more importantly the type of fat accumulation. Physical activity reduces the visceral fat tissue, which is an important factor in the development of MetS [35]. Also, studies have indicated the preventive effect of exercise on dyslipidemia, inflammation, and blood pressure [35, 36].

In the present study, an ethnospecific approach was used in determining the waist circumference component of MetS [3]. A study in Canada [37] used the Adult Treatment Panel III criteria [38] and found a prevalence of 17.7% among adult Canadians, which seems to be similar compared to our prevalence of 16.9% for Canadian adolescent and adults. Different ethnic groups with the same measures of central obesity are shown to have a different risk of CVD and diabetes [39]. However, other ethnospecific cutoffs are needed for the other MetS components such as the blood pressure component.

### 4.3. Association between Metabolic Syndrome and Dietary Patterns

The “fast-food” dietary pattern observed among 50–79-year-old Canadians was associated with MetS in our study, which is in agreement with results from other studies [5, 7, 13]. The reason for not observing this significant association among adolescents and younger adult Canadians maybe related to the lower prevalence of MetS in these younger age groups. The foods that had a positive loading on the “fast-food” dietary pattern in our study have shown to increase the risk of different components of MetS in other studies. This dietary pattern included processed meat, which similarly was found to increase the risk of MetS in two studies conducted in Iran [40] and the United States [41]. Researchers indicate that the heme-iron, saturated/trans-fatty acids, nitrites, and advanced glycation end products are contributors to the increased risk of MetS [13, 40]. Similar to other studies conducted in the United States, chips and fries as two high-fat, processed foods were observed to have high loadings on the “fast-food” dietary pattern of the older adult population of our study [42]. Importantly, the partially hydrogenated vegetable oils content of fries contributes to an increased trans-fatty acid content of the diet which in return increases systemic inflammation, endothelial dysfunction, and insulin resistance [43]. In the present study, diet soft drinks were found to be consumed more among people with MetS compared to ones without MetS (ages 12–79 years). Similarly, other studies have found diet soft drinks to be associated with the risk of MetS, among European and American populations [44, 45]. Reverse causality, confounding the effect of other factors, and the presence of an underlying pathophysiological effect of an ingredient of the diet soft drinks are the reasons under discussion for this finding [44, 45].

The “healthy-like” dietary patterns that emerged in our study in different age groups were characterized by high nutrient-dense foods. However, in agreement with our results, many other studies had not observed an association between this dietary pattern and MetS [7]. There are different components of the “healthy-like” dietary patterns, which are not similar in all studies. As well, MetS has different components and features. Thus, MetS prevention/reversal requires a dietary pattern that could affect all features of a MetS at the same time such as the Mediterranean diet or the Dietary Approaches to Stop Hypertension [8, 9, 11].

Our study is the first in Canada to investigate the association of MetS and dietary patterns among Canadians 12–79 years of age. We used nationally representative objective health measures data from CHMS, collected from Canadians for the first time. Another strength of this study is the dietary pattern approach used, which illustrated a holistic picture of the dietary patterns prevalent among Canadians, and since our outcome is a multifeatured syndrome, a dietary pattern approach is more suitable for this outcome.

A limitation of our study was the food frequency questionnaire used in the analysis, which does not include a few foods from grains and meat and alternatives groups [19]. Furthermore, our study is a cross-sectional study; thus, causality cannot be concluded, and there are chances of reverse causality due to the nature of cross-sectional studies. In addition, the alpha posteriori method includes researcher-oriented decisions and, thus, may reduce the reproducibility of the analysis. Finally, we have adjusted for many sociodemographic and lifestyle factors; however, there may still be unobserved differences leading to residual confounding.

## 5. Conclusions

The growing prevalence of obesity, metabolic abnormalities and MetS, and having a “fast-food” dietary pattern, which is associated with 27% increased risk of MetS among older Canadians, is important in terms of policy implications in the area of nutrition and health. The association between MetS and the “fast-food” dietary pattern remained significant after adjustment for all sociodemographic/lifestyle variables suggesting the importance of diet quality/composition. However, further investigations using prospectively designed studies are needed across the lifespan.

## Figures and Tables

**Table 1:** Prevalence (weighted estimate) of metabolic syndrome across sociodemographic and lifestyle factors of Canadians aged 12 to 79 years, Canadian Health Measures Survey combined Cycles 1 and 2, 2007–2011.

	Metabolic syndrome, estimated prevalence % (SE)	95% CI	*p* value^a^
*Age (years)*			
12–19^*b*^	2.4 (0.8)^*c*^	0.7–4.1	—
20–49	11.5 (1.3)	8.8–14.1	0.002
50–79	30.1 (2.2)	25.7–34.6	<0.001
*Sex*			
Males	18.1 (1.4)	15.2–21	0.288
Females^*b*^	15.8 (1.7)	12.3–19.3	—
*Income*			
Lowest	14.5 (3.4)^*c*^	7.5–21.6	1.000
Lower middle	25.8 (3.3)	19.1–32.6	0.031
Upper middle	17.3 (1.7)	13.9–20.8	0.519
Highest^*b*^	14.1 (1.6)	10.7–17.5	—
*Education* ^*e*^			
<Secondary	36.2 (7.1)^*c*^	21.5–51.0	0.014
Secondary grad.	22.7 (3.4)	15.6–30.0	0.075
Some postsecondary	12.2 (2.9) ^*c*^	6.3–18.1	0.952
Postsecondary grad.^*b*^	14.2 (1.1)	12.0–16.4	—
*Alcohol*			
Ever	*d*	12.5–25.0	0.471
Never^*b*^	16.5 (1.2)	14.0–19.0	—
*Physical activity*			
Active^*b*^	9.3 (1.1)	7.0–11.5	—
Moderately active	18.7 (2.2)	14.2–23.3	0.002
Inactive	19.8 (1.8)	16.2–23.5	<0.001
*Smoking*			
Current	18.6 (2.6)	13.2–24.0	0.131
Former	24.0 (2.2)	19.5–28.6	0.002
Never^*b*^	13.1 (1.4)	10.2–15.9	—
*Family doctor*			
Yes	18.2 (1.3)	15.6–20.9	0.022
No^*b*^	11.0 (2.1) ^*c*^	6.5–15.4	—

Table sample includes *n* = 4,272 (males = 49.6%), representative of 26,038,108 Canadians aged 12 to 79 years. CI: confidence interval, SE: standard error. aBinary logistic regression, *α* = 0.05 significant level. bReference level. cCoefficient of variation 16.6% to 33.3%, recommended to be used with caution.dCoefficient of variation >33.3%, unreliable for publishing based on Statistics Canada's recommendation. eTotal household income; lowest income < $15,000 if 1/2 people or <$30,000 if more than 4 people in the household. The lower-middle income level included those having a total income of $15,000–$29,999 if 1/2 people; $20,000–153 $39,999 if 3/4 people; and $30,000–$59,999 if more than four people were living in the household. The upper-middle category indicated an income of $30,00–$59,999 if 1/2 people; $40,000–$79,999 if 3/4 people; and $60,000–$79,999 if there were more than four in the household. The highest-level income included those with an income of $60,000 or more if 1/2 people and an income of $80,000 if more than two people were living in the household [14].

**Table 2:** Factor loadings from principal component analysis of dietary intake of Canadians 12–79 years of age, Canadian Health Measures Survey combined Cycles 1 and 2, 2007–11.

Food/food groups^a^	Dietary patterns for age 12–19 y				Dietary patterns for age 20–49 y				Dietary patterns for age 50–79 y			
	F1^b^	F2	F3	F4	F1	F2	F3	F4	F1	F2	F3	F4
Red meat	0.39^†^	—	—	0.40	0.59	—	—	—	—	—	—	0.72
Liver meat	—	—	—	—	—	—	—	0.83	—	—	—	—
Other than liver organ meat	—	—	—	0.42	—	—	—	0.84	—	—	—	—
Hot dogs	0.57	—	—	—	0.46	—	—	—	—	—	0.55	.
Sausage/bacon	0.59	—	—	—	0.59	—	—	—	—	—	0.29	0.55
Beans	—	—	—	0.25	—	0.24		—	—	—	—	—
Nuts	—	—	—	0.44	—	0.36	0.26	—	0.38	—	—	—
Eggs	—	—	—	0.59	0.35			—	—	—	—	0.38
Fruit	—	0.68	—	—	—	0.45	0.40	—	0.36	—	—	—
Tomato and tomato sauce	—	0.25	0.27	—	—	0.50	—	—	—	0.58	—	—
Lettuce/green vegetables	—	0.22	0.74	—	—	0.75	—	—	0.28	0.73	—	—
Spinach/mustard greens/collards	—	—	0.44	0.29	—	0.50	—	—	0.41	0.22	—	—
Baked/boiled/mashed potatoes	0.24	—	—	—	0.47	—	—	—	—	—	—	0.63
Other vegetables	−0.22	0.60	0.25	—	—	0.45	0.24	—	0.50	—	—	—
Milk	—	—	—	—	—	—	0.73	—		—	—	—
Cheese	—	—	—	—	—	0.21		—	0.33	—	—	—
Yogurt	—	0.56	—	—	—	0.28	0.47	—	0.48	—	—	—
Ice cream/frozen yogurt	—	—	—	—	—	—	—	—	—	—	—	0.30
Salad dressing/mayonnaise	—	—	0.72	—	0.22	0.58	—	—	—	0.72	—	—
Chips	0.29	—	—	—	0.46	—	—	—	—	—	0.62	—
Fries	0.57	—	—	—	0.57	—	—	—	—	—	0.61	0.20
Brown bread	—	—	—	—		—	—	—	—	—	—	—
White bread	—	—	—	—	0.25	—	—	—	—	—	—	0.23
Rice	—	—	—	0.64		—	—	—	—	—	—	—
Pasta	0.21	—	—	—	0.36	—	—	—	—	—	0.21	—
Cereal	—	—	—	—		—	0.73	—	—	—	—	—
Diet soft drinks	0.46	0.33	—	−0.20	0.32	—	—	—	0.28	—	0.57	—
Regular soft drinks	0.32	−0.30	—	—	—	—	—	—	0.58	—	—	—
Sport drinks	0.37	—	—	—	—	—	—	—	—	—	—	—
Flavoured drinks	—	—	—	—	—	—	—	—	—	—	—	—
Fruit juice	—	0.39	—	—	—	—	—	—	—	—	—	—
Vegetable juice	—	—	—	—	—	—	—	—	0.31	—	—	—

Sample includes *n* = 4,272 participants. Only factor loading scores below −0.2 and above 0.2 are shown in this table.^a^Detailed description of the food/food groups are indicated in supplementary file, Table S1.^†^F1–4 are dietary patterns 1–4. For ages 12–19 y, F1: “Western”; F2: “healthy-like”; F3: “salad and condiments”; F4: “protein/rice.” For ages 20–49 y, F1: “Western”; F2: “healthy-like”; F3: “nuts, fruits and vegetables, dairy, and cereal”; F4: “organ meat.” For ages 50–79 y, F1: “healthy-like”, F2: “salad and condiments”; F3: “fast food”; F4: “meat and potato.”

**Table 3:** Multivariate-adjusted odds ratios for the association between metabolic syndrome and dietary patterns (factors) by age group, Canadian Health Measures Survey combined Cycles 1 and 2, 2007–11.

Age group	Factors^a^	Model^b^	Odds ratio	LCI^c^	UCI^d^	*p* value
12–19 y	F1	Model 1	2.29	0.58	9.04	0.236
		Model 2	2.36	0.09	60.83	0.604
	F2	Model 1	0.50	0.12	2.03	0.331
		Model 2	0.28	0.00	50.07	0.634
	F3	Model 1	0.82	0.37	1.82	0.621
		Model 2	0.78	0.09	6.91	0.830
	F4	Model 1	1.18	0.39	3.61	0.772
		Model 2	0.60	0.01	38	0.813

20–49 y	F1	Model 1	1.02	0.73	1.44	0.900
		Model 2	1.09	0.75	1.58	0.656
	F2	Model 1	0.79	0.59	1.06	0.110
		Model 2	0.83	0.61	1.14	0.255
	F3	Model 1	0.77	0.59	1.01	0.063
		Model 2	0.83	0.60	1.15	0.259
	F4	Model 1	1.21	0.88	1.65	0.244
		Model 2	1.14	0.74	1.75	0.552

50–79 y	F1	Model 1	0.86	0.66	1.11	0.233
		Model 2	0.90	0.69	1.17	0.438
	F2	Model 1	0.89	0.72	1.10	0.275
		Model 2	0.91	0.74	1.13	0.403
	F3	Model 1	1.25	1.03	1.51	0.021
		Model 2	1.27	1.04	1.54	**0.020**
	F4	Model 1	1.03	0.87	1.22	0.723
		Model 2	1.05	0.88	1.25	0.578

Sample included in the analysis for this table includes 4,272 participants (males = 49.6%) representative of 26,038,108 Canadians aged 12 to 79 years.^a^F1–4 are dietary patterns 1–4. For ages 12–19 y, F1: “Western”; F2: “healthy-like”; F3: “salad and condiments”; F4: “protein/rice.” For ages 20–49 y, F1: “Western”; F2: “healthy-like”; F3: “nuts, fruits and vegetables, dairy, and cereal”; F4: “organ meat.” For ages 50–79 y, F1: “healthy-like”, F2: “salad and condiments”; F3: “fast food”; F4: “meat and potato.” ^b^Model 1 adjusted for age and sex; model 2 adjusted for age, sex, income, education, physical activity, alcohol intake, and other dietary patterns between 1–4. ^c^Lower 95% confidence interval.^d^Upper 95% confidence interval.

## Data Availability

The CHMS data used in this study were provided by the Research Data Center-Statistics Canada. Due to confidentiality, requests for access to these data should be made to the Research Data Center at the University of Saskatchewan, Saskatoon, Canada.

## Supplementary Materials

Supplementary Materials Supplementary file 1 is provided for this manuscript cited in the text. This file contains Table S1, which is “grouping of CHMS dietary intake questions”; Figure S1 which is “sample selection from fasted combined Cycles 1 and 2 of the Canadian Health Measures Survey 2007–11”; and Table S2 that includes “food intake of Canadians aged 12 to 79 years with and without Metabolic Syndrome (MetS), Canadian Health Measures Survey combined Cycles 1 and 2, 2007–2011 (*n* = 4,272, males = 49.6%, representative of 26,038,108 Canadians aged 12 to 79 years).”
