# Supplementary material for: Fast-Food Dietary Pattern Is Linked to Higher Prevalence of Metabolic Syndrome in Older Canadian Adults
Source: J Nutr Metab. 2021 Oct 21;2021:5712844. doi: 10.1155/2021/5712844 (PMC8553465; doi:10.1155/2021/5712844)
Supplement: Supplementary Materials — Supplementary file 1 is provided for this manuscript cited in the text. This file contains Table S1, which is “grouping of CHMS dietary intake questions”; Figure S1 which is “sample selection from fasted combined Cycles 1 and 2 of the Canadian Health Measures Survey 2007–11”; and Table S2 that includes “food intake of Canadians aged 12 to 79 years with and without Metabolic Syndrome (MetS), Canadian Health Measures Survey combined Cycles 1 and 2, 2007–2011 (n = 4,272, males = 49.6%, representative of 26,038,108 Canadians aged 12 to 79 years).” [file 5712844.f1.doc]

Table S1. Grouping of CHMS dietary intake questions.

| **Food groups^1^** | **CHMS food frequency questionnaire items^2, 3^,** |
| --- | --- |
| **CHMS Questions on Meat and Alternatives group** | -Beef or pork hot dogs  -Cooked dried beans, such as refried beans, baked beans, pea soup or kidney beans, excluding green and yellow beans  -Eggs and egg dishes including the yolk (excluding all egg dishes made with only egg whites); egg dishes could include eggs, omelettes, frittata or quiche.  -Fish- and shellfish-related questions in Cycle 2 [3]  -Liver (including all types of liver such as beef, veal, pork or chicken, but excluding liverwurst and liver pâté)  -Other organ meats such as kidneys, heart or giblets  -Peanuts, walnuts, seeds, or other nuts, excluding nut butters such as peanut butter  -Red meat (beef, hamburger, pork or lamb)  -Sausage or bacon (including all types of sausage, such as breakfast, pepperoni and kielbasa but excluding low-fat, light or turkey varieties) |
| **CHMS questions on Milk and Alternatives group** | -Cottage cheese  -Milk or enriched milk substitutes. Questions are asked about the kinds of milk usually consumed (3.25, 1, 0.5, skim or non-fat), flavoured milk beverages (chocolate milk and flavoured milk beverages such as Oh Henry®, rice, soya and other).  -Yogurt, excluding frozen yogurt  -Ice cream or frozen yogurt |
| **CHMS questions on Grain products group** | -Any kind of pasta (including spaghetti, noodles, macaroni & cheese or pasta salad)  -Any kind of rice  -Brown bread, including bagels, rolls, pita bread or tortillas  -Hot or cold cereal  -Instant, seasoned or wild rice (such as Minute Rice®, Dainty®, Rice-a-Roni®)  -White bread, including bagels, rolls, pita bread or tortillas |
| **CHMS questions on Vegetables and Fruit group** | -Fruit (fresh, frozen or canned)  -Lettuce or green leafy salad with or without other vegetables  -Other than French fries, home fries, or hash brown potatoes, including baked, boiled, mashed or in potato salad, but excluding sweet potatoes  -Spinach, mustard greens or collards excluding kale  -Tomatoes or tomato sauce, including salsa, tomato soup and spaghetti sauce but excluding tomato paste, ketchup or pizza sauce  -French fries, home fries, or hash brown potatoes  -Fruit and vegetables juice (includes fruit juice and vegetables juice)  -All other types of vegetables excluding those already mentioned |
| **CHMS questions on dietary fat** | -Regular-fat potato chips, tortilla chips or corn chips (excluding low fat chips and pretzels).  -Regular-fat salad dressing or mayonnaise (including on salads and sandwiches) |
| **CHMS questions on beverages** | -Diet soft drinks  -Sugar sweetened beverages (includes fruit flavoured drinks, regular soft drinks, sport drinks, such as Gatorade® or PowerAde®)  -Fruit and vegetables juice (includes fruit juice and vegetables juice) |

CHMS: Canadian Health Measures Survey

^1^ Food groups are named based on the 1992 Canada’s Food Guide and reported in Garriguet (2007).

^2^ The question asked for each category was as follows, “how often do you usually eat per day/week/month/year?”, otherwise the whole question is asked (Statistics Canada 2010, 2012).

1. Garriguet D. 2007. Canadians' eating habits. Health Rep, 18(2):17.
2. Statistics Canada. 2010. Canadian Health Measures Survey – Data Dictionary: Cycle 1. Ottawa. Available at <http://www.library.carleton.ca/sites/default/files/find/data/surveys/pdf_files/chms-c1-07-09-dic.pdf> [Accessed 10 October 2019.]
3. Statistics Canada. 2012. Canadian Health Measures Survey (CHMS) Data User Guide: Cycle 2, Ottawa. Available at Research Data Center upon request.

**Figure S1**. Sample selection from fasted combined Cycles 1 and 2 of the Canadian Health Measures Survey 2007-11.

Cycle 1

N= 5 600

6-79y

Cycle 2

N= 6 400

3-79y

Combined Cycles 1 & 2 fasted sub-sample 6-79y

N= 5 427

Combined Cycles 1 & 2 fasted sub-sample

non-pregnant and not diagnosed with diabetes 12-79y

N= 4 272

representative 26,038,100 Canadians.

Table S2. Food intake of Canadians aged 12 to 79 years with and without Metabolic Syndrome (MetS), Canadian Health Measures Survey, combined Cycles 1 and 2, 2007–2011 (n=4,272, males=49.6%, was representative of 26,038,108 Canadians aged 12 to 79 years).

| **Food/food groups** | **Without MetS**  **Mean (SE) (95% CI)** | **With MetS**  **Mean (SE) (95% CI)** | | |
| --- | --- | --- | --- | --- |
| ***Meat and alternatives*** | | | | |
| Red meat | 161.68 ± 2.93  (155.62-167.74) | | | 158.28 ± 7.06  (143.70-172.86) |
| Liver | - 1. ± 0.45   (2.56-4.44) | | | 5.03 ± 0.62  (3.75-6.31) |
| Other organ meat such as kidneys, heart or giblets | 1.78 ± 0.37  (1.02-2.54) | | | 1.25 ± 0.30  (0.64-1.87) |
| Hotdogs | 18.39 ± 1.50  (15.30-21.47) | | | 16.34 ± 1.96  (12.30-20.39) |
| Sausage | 40.00 ± 1.91  (37.05 -44.94) | | | 39.02 ± 4.16  (30.42-47.61) |
| Eggs/egg dishes | **104.94** ± **2.69***  **(99.39-110.49)** | | | **93.01** ± **3.27**  **(86.26-99.76)** |
| Beans | 52.76 ± 3.79  (44.93-60.59) | | | 40.44 ± 3.51  (33.19-47.70) |
| Nuts (excluding nut butters) | **102.78** ± **4.22**  **(94.07-111.49)** | | | **79.70** ± **4.60**  **(70.20-89.21)** |
| ***Dairy*** | | | | |
| Milk | 353.68 ± 11.92  (329.08-378.27) | 295.85 ± 20.62  (253.31-338.40) | | |
| Cheese | 13.98 ± 1.13  (11.65-16.30) | 16.79 ± 2.30  (12.05-21.53) | | |
| Yogurt | 137.07 ± 5.05  (126.66-147.48) | 131.10 ± 9.21  (112.09-150.11) | | |
| **Grains** | | | | |
| Cereal (hot or cold) | 153.50 ± 3.94  (145.35-161.64) | 152.35 ± 16.43  (118.43-186.26) | | |
| Brown bread | 244.63 ± 5.970  (232.31-256.95) | 227.13 ± 13.25  (199.78-254.48) | | |
| White bread | 136.33 ± 5.87  (124.22-148.45) | 155.29 ± 26.07  (101.48-209.11) | | |
| Pasta | **97.40** ± **1.586**  **(94.13-100.67)** | **72.94** ± **4.25**  **(64.18-81.71)** | | |
| Rice | 115.97 ± 8.05  (99.36-132.58) | 97.04 ± 9.36  (77.72-116.37) | | |
| ***Fruits and vegetables*** | | | | |
| Fruits | 460.62 ± 12.24  (435.37-485.88) | | 430.81 ± 22.59  (384.18-477.45) | |
| Tomato/tomato sauce | 145.00 ± 3.98  (136.78-153.21) | | 149.37 ± 8.75  (131.31-167.44) | |
| Lettuce/green leafy salads | 160.20 ± 4.14  (151.65-168.74) | | 146.72 ± 8.23  (129.74-163.70) | |
| Spinach/mustard greens/collards | 46.35 ± 4.31  (37.45-55.25) | | 33.32 ± 3.36  (26.38-40.25) | |
| Fries (any type including hash brown) | 54.46 ± 2.60  (49.09-59.84) | | 47.10 ± 3.93  (38.99-55.20) | |
| Baked/boiled/mashed potatoes | 94.95 ± 3.97  (86.76 -103.16) | | 114.08 ± 8.65  (96.23-131.94) | |
| Other [than mentioned] vegetables | 350.20 ± 6.78  (336.21-364.20) | | 314.39 ± 24.36  (264.12-364.67) | |
| ***Dietary fat*** | | | | |
| Dressing/mayonnaise | 121.027 ± 4.07  (112.63-129.42) | | 106.61 ± 6.94  (92.29-120.92) | |
| Chips (any type excluding low-fat and pretzels) | 59.28 ± 2.61  (53.89-64.68) | | 46.58 ± 4.32  (37.66-55.49) | |
| ***Beverages*** | | | | |
| Diet soft drinks | **44.35**  ± **4.68**  **(34.70-54.00)** | | **81.10**  ± **12.63**  **(55.02-107.17)** | |
| Regular soft drinks | 93.92 ± 7.33  (78.79-109.05) | | 98.58 ± 13.60  (70.51-126.65) | |
| Sport drinks | **21.53**  ± **1.27**  **(18.92 -24.15)** | | **8.92**  ± **2.08**  **(4.63-13.21)** | |
| Flavoured drink | 50.87 ± 3.10  (44.46 -57.28) | | 39.65 ± 7.23  (24.73-54.57) | |
| Fruit juice | 209.04 ± 7.03  (194.53-223.56) | | 201.98 ± 16.51  (167.91-236.05) | |
| Vegetable juice | 32.06 ± 2.50  (26.90-37.21) | | 41.95 ± 6.72  (28.08-55.82) | |

CI: Confidence interval; MetS: metabolic syndrome.

* Values in bold indicate significant difference in the row based on 95% confidence interval non-overlap.
